# Case report: Successful remission with upadacitinib in a young patient with anti-TNF-refractory intestinal Behçet’s disease

**DOI:** 10.3389/fimmu.2024.1483993

**Published:** 2024-11-08

**Authors:** Sumei Sha, Bin Xu, Kairuo Wang, Chenyang Qiao, Haitao Shi, Jiong Jiang, Xiaojing Quan, Xin Liu

**Affiliations:** ^1^ Department of Gastroenterology, The Second Affiliated Hospital of Xi'an Jiaotong University, Xi’an, Shaanxi, China; ^2^ Department of Radiotherapy, Tangdu Hospital of the Air Force Medical University, Xi’an, Shaanxi, China

**Keywords:** intestinal Behçet’s disease, upadacitinib, case report, mucosal healing, anti-TNF-refractory

## Abstract

**Background:**

The limited therapeutic options and inconsistent treatment efficacy of Intestinal Behçet’s disease complicate its management, with the absence of standardized guidelines further exacerbating the challenges faced by clinicians.

**Case Presentation:**

In this case report, we present a patient with refractory intestinal Behçet’s disease who experienced treatment failure with prior biologic agents. This case sheds light on managing this complex scenario with Janus kinase (JAK) inhibitors, the patient achieved mucosal healing after switching to Upadacitinib.

**Conclusion:**

This case underscores the potential of Upadacitinib as an effective alternative for managing difficult cases of intestinal Behçet’s disease.

## Introduction

Intestinal Behçet’s disease is a rare autoimmune disorder characterized by recurrent intestinal ulcers, abdominal pain, and diarrhea. Traditional treatment options include corticosteroids, immunosuppressants, and biologic agents (1). However, due to the complex etiology and pathophysiology of this condition, many patients do not respond adequately to traditional therapies, resulting in cases that are classified as refractory (2). In recent years, upadacitinib, a novel Janus kinase (JAK) inhibitor, has demonstrated promising potential in the treatment of various autoimmune diseases. Its mechanisms of action involve the modulation of inflammatory pathways and it is currently employed in the management of inflammatory bowel disease, rheumatoid arthritis, ankylosing spondylitis, and psoriasis, among other autoimmune-related inflammatory conditions (3, 4). This emerging treatment option offers new hope for patients with challenging cases who have previously experienced limited success with standard therapies. Here, we present a male patient in whom upadacitinib successfully induced remission of Intestinal Behçet’s disease in steroid-dependent and resistant to anti-TNF-α. To the best of our knowledge, this is the first report of upadacitinib administration in childbearing age male patient of Intestinal Behçet’s disease, highlighting its potential role in managing this challenging condition and offering new hope for affected patients.

## Case presentation

**Figure 1:**
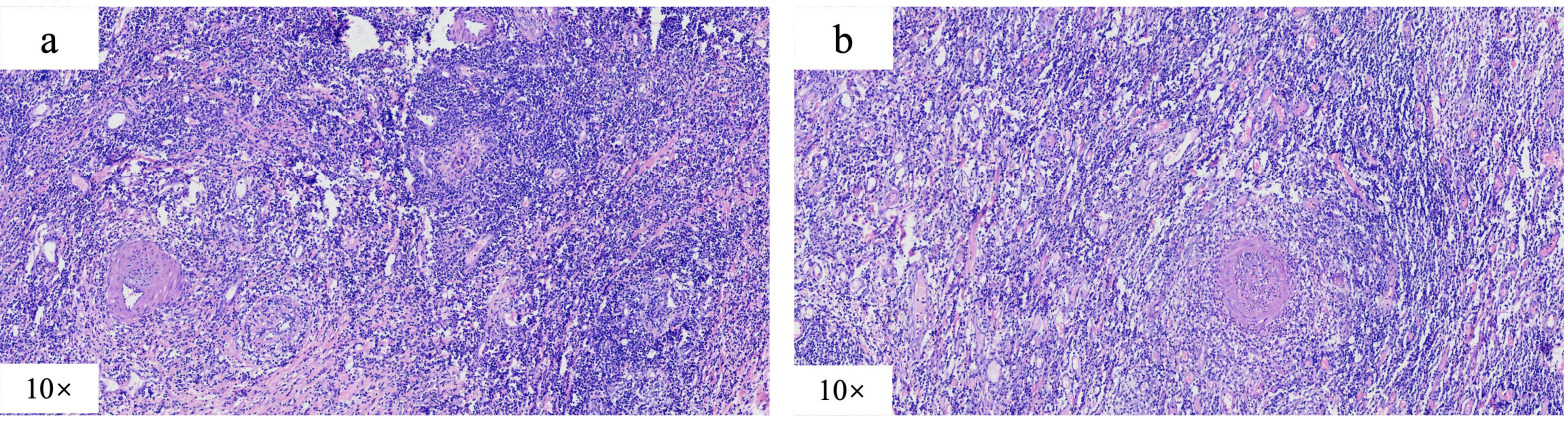
Postoperative pathological manifestations, which indicated multiple occlusive small vein inflammations and lymphoid tissue aggregation were found on the mesentery side of the (**A**) cecum and ascending (**B**) colon.

**Figure 2:**
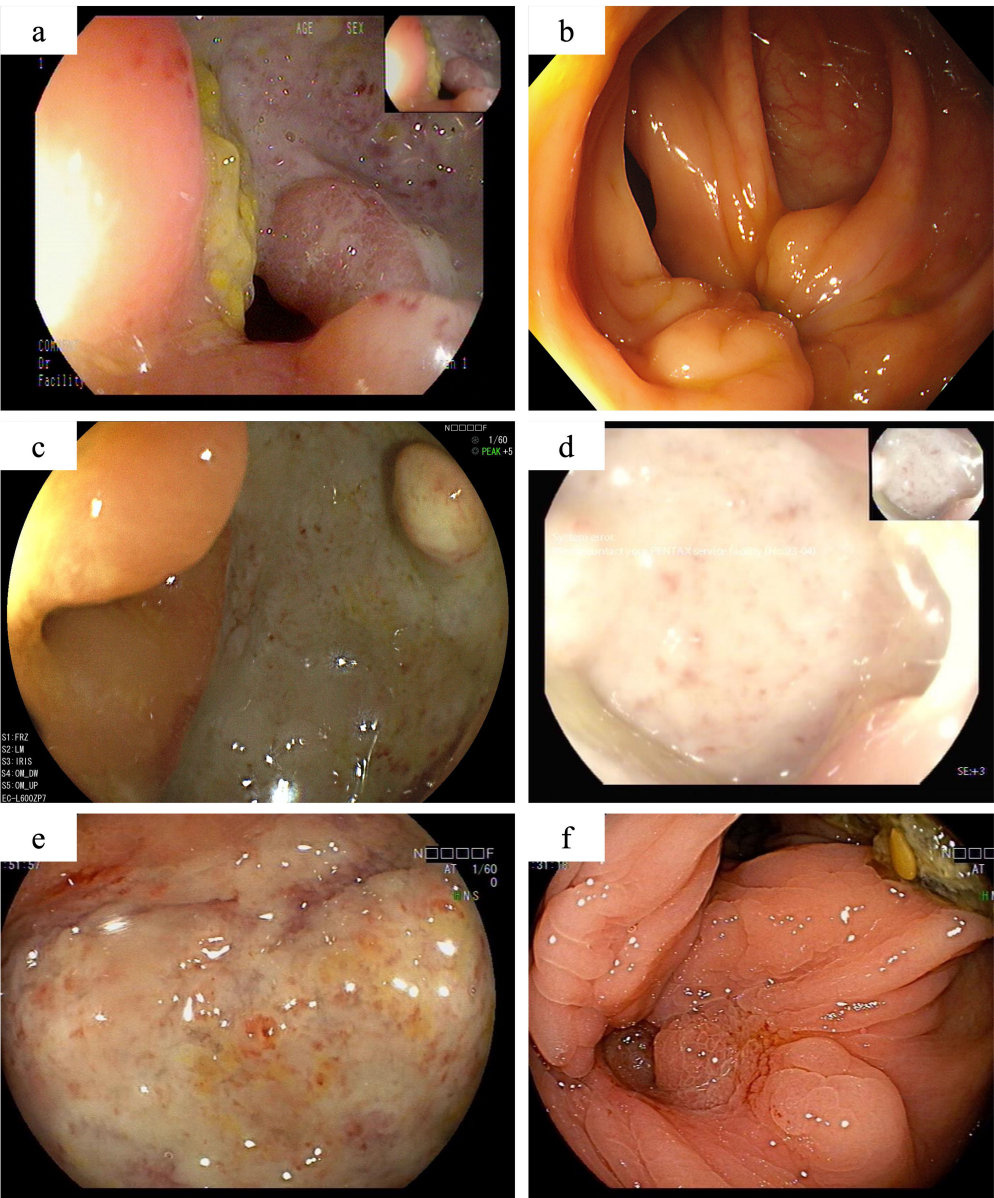
The colonoscopy findings of this patient. One year after partial colonic resection, colonoscopy demonstrated a large, well-defined round ulcer adjacent to the anastomosis site **(A)**. Scar formation and mucosal aggregation can be seen near the anastomotic site with no ulcers are observed after induction treatment with Infliximab **(B)**. Secondary unresponsive to Infliximab **(C)**, with no significant improvement after intensified treatment. Colonoscopy revealed the appearance of large ulcers around the anastomotic site again **(D)**. When switching to adalimumab combined with sulfasalazine treatment, there is still no sign of improvement in ulcers near the anastomotic site **(E)**. After 12 weeks of oral administration of upadacitinib, colonoscopy showed complete healing of the ulcer **(F)**.

**Figure 3:**
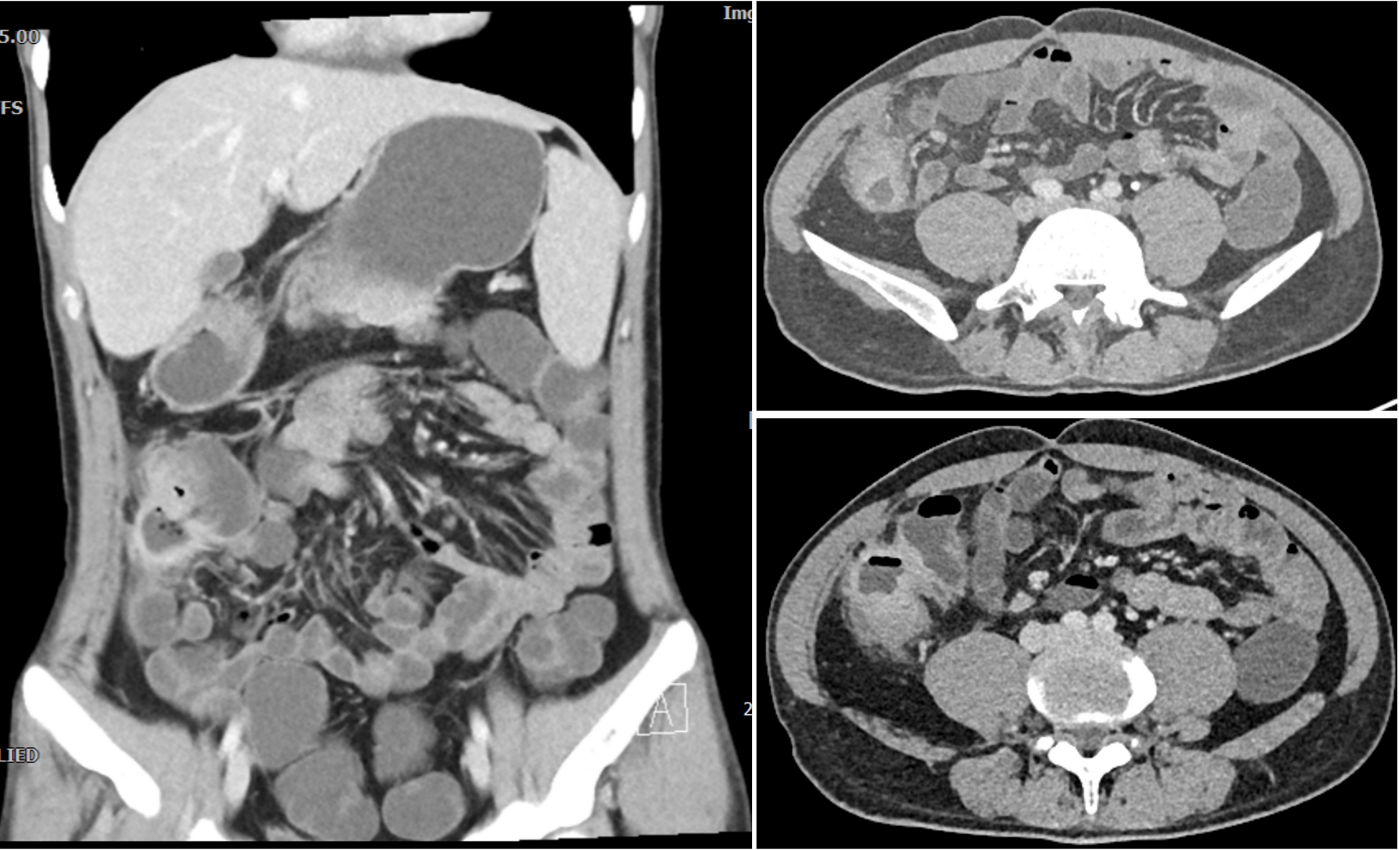
CTE manifestations of patients with recurrent abdominal pain one year after surgery. It revealed significant uneven thickening of the intestinal wall in the lower right abdomen, obvious extravasation outside the serosa, and multiple small vessel shadows, which were significantly enhanced after enhancement.

We present the case of a 24-year-old male diagnosed with intestinal Behçet’s disease. Four years prior, he experienced recurrent fever, oral ulcers and abdominal pain. Colonoscopy revealed a single, well-defined large ulcer in the ileocecal region. Despite undergoing diagnostic anti-tuberculosis treatment, his symptoms and ulcers did not improve. On August 13, 2021, the patient underwent laparoscopic partial colonic resection. Post-operative pathology indicated multiple occlusive small vein inflammations and lymphoid tissue aggregation were found on the mesentery side of the cecum and ascending colon (**Figure 1**). ultimately resulting in a diagnosis of intestinal Behçet’s disease, although no medical therapy was initiated at that time. One year after surgery, the patient was readmitted due to recurrence of right lower abdominal pain. Laboratory tests indicated significant inflammatory markers (c reactive protein=67.8mg/L, erythrocyte sedimentation rate=29mm/h, faecal calprotectin=989.10ug/G), along with anemia, while colonoscopy demonstrated a large, well-defined round ulcer adjacent to the anastomosis site (**Figure 2A**). Both ANAs (such as anti-dsDNA, nucleosome, histone, Sm, SS-A/Ro52, SS-A/Ro60, SS-B/La, CENP-B, Scl-70, AMA-M2, Jo-1, ribosomal-P and RNP) and ANCA are negative. The CTE showed significant uneven thickening of the intestinal wall in the lower right abdomen, obvious extravasation outside the serosa, and multiple small vessel shadows, which were significantly enhanced after enhancement (**Figure 3**).

In the initial treatment phase, systemic corticosteroids were administered to quickly alleviate inflammation, and infliximab therapy was initiated as corticosteroid dose reduction began. After three months of treatment, the corticosteroids were successfully tapered, and the patient continued regular infliximab therapy. At the end of the induction phase, he reported significant relief from abdominal pain, normalization of inflammatory markers, and complete healing of the ulcers on follow-up endoscopy (**Figure 2B**). However, eight months later, the patient again experienced abdominal pain with colonoscopy revealing a recurrence of anastomotic ulcers (**Figure 2C**). Therapeutic drug monitoring (TDM) indicated an infliximab level of 4.3 μg/ml without the presence of anti-drug antibodies. Consequently, intensification of infliximab therapy with shortened intervals (from every 8 weeks to every 4 weeks) was recommended, but his condition did not improve (**Figure 2D**).

Subsequently, the patient underwent short-term systemic corticosteroid treatment before switching to a regimen of sulfasalazine combined with adalimumab in the rheumatology department to induce remission. Unfortunately, this treatment proved ineffective, with persistent abdominal pain, elevated inflammatory markers, and no significant improvement in colonoscopy findings (**Figure 2E**). After discussing the potential benefits and risks of alternative therapies, the patient and his parents consented to initiate upadacitinib treatment. Therefore, adalimumab was discontinued, and on February 21, 2024, the patient commenced treatment with 45 mg of upadacitinib daily. His abdominal pain symptoms rapidly improved, with no more oral ulcers nor fever appeared, inflammation indicators (c reactive protein, erythrocyte sedimentation rate and faecal calprotectin) returned to normal, anemia was corrected, and stool routine examination showed negative. By the 12-week follow-up, complete ulcer healing was noted (**Figure 2F**). The patient’s condition has remained stable, allowing him to return to normal daily activities, and the dosage of upadacitinib has been tapered to 30 mg for maintenance therapy. The patient experienced significant symptomatic improvement and a notable enhancement in quality of life, without the occurrence of severe adverse events up to now.

## Discussion

Intestinal Behçet’s disease is a severe condition that significantly impacts patients’ quality of life due to its complex and often debilitating manifestations. Particularly when affecting the intestines, it presents significant therapeutic challenges due to the heterogeneous nature. Systemic glucocorticoids paired with other immunosuppressive agents, constitute the first line of treatment for Intestinal Behçet’s disease currently. In particular, the addition of anti-tumor necrosis factor inhibitors (anti-TNF) further controlled Behcet’s disease (5). Among them, corticosteroids can provide rapid symptom relief, their long-term use is often associated with a broad spectrum of adverse effects, including osteoporosis, cardiovascular issues, and metabolic disturbances. Immunosuppressants like azathioprine and methotrexate provide therapeutic benefits; however, concerns regarding their side effects and potential reproductive risks have led to a cautious approach. About 30% of such patients respond inadequately to treatment with anti-TNF while presenting with refractory and relapse inflammation (6). Additionally, issues like secondary loss of response further complicate their use, underscoring the urgent need for novel therapeutic strategies that can more effectively manage this condition. This variability emphasizes the need for personalized approaches to optimize therapeutic outcomes.

The intricate pathogenesis of Intestinal Behçet’s disease suggests a complex interplay of multiple immune pathways. Recent evidence suggests that the Janus kinase (JAK) signaling pathway may play a role in the disease process, with the JAK-STAT pathway emerging as a potential therapeutic target (7). Previous study (8) demonstrated the role of the JAK/STAT pathway in peripheral blood CD14+ monocyte and CD4+ T cells with whole genome gene expression analysis and confirmed our results with flow cytometric STAT3 analysis in peripheral blood of patients with Behçet’s disease. This hypothesis is reinforced by the successful application of JAK inhibitors, notably tofacitinib, a non-selective JAK inhibitor, which has demonstrated clinical and laboratory improvements in Intestinal Behçet’s disease patients (9, 10). Through inhibition of JAK, upadacitinib inhibits phosphorylation of downstream effector proteins, which consequently inhibits cytokine signaling for key pathways involved in inflammatory diseases (3). Furthermore, the JAK-1 targeting upadacitinib has been approved for the treatment of rheumatoid arthritis, psoriatic arthritis, ankylosing spondylitis, and inflammatory bowel disease (11). However, data regarding the use of selective JAK inhibitors, like upadacitinib, in this context remain scarce, presenting an area of active investigation.

Recent case reports have highlighted the efficacy and safety of upadacitinib in patients with systemic Behçet’s disease associated with other complications such as ankylosing spondylitis and uveitis (12, 13). However, no data is yet available on upadacitinib use in patients with intestinal Behçet’s disease in childbearing age. Our case report highlights the promising role of upadacitinib in a young patient with refractory intestinal Behçet’s disease, who had failed previous therapies including infliximab, sulfasalazine, and adalimumab. The favorable outcomes observed in this patient, along with previous case reports documenting the efficacy and safety of upadacitinib in systemic Behçet’s disease patients with comorbid conditions like ankylosing spondylitis and uveitis, suggest that upadacitinib has emerged as a promising treatment for refractory intestinal Behçet’s disease, particularly for patients who have not responded adequately to biologic therapies (**Table 1**). At the same time, previous reports suggest that upadacitinib may increase the risk of acne, herpes zoster, non-melanoma skin cancer, elevations in creatine phosphokinase levels, serious infections, major adverse cardiovascular events, venous thromboembolism and malignancies (14). We need to be vigilant about the safety issues of long-term use.

**Table 1:** Case reports of successful remission with upadacitinib in patients with Behçet’s disease.

Country	Years, Sex	Complication	Previous treatments	Dosage of	Clinical outcome
Bulgaria (12)	42, female	ankylosing spondylitis	corticosteroids	15mg	signs having undergone substantial improvement or achieving complete resolution
China (13)	adolescent girl	Macular Edema, Behçet’s Uveitis	topical antiinflammatory drops, systemic corticosteroids, methotrexate, cyclosporine A, adalimumab and mycophenolate mofetil	15mg	symptom-free for five months and has been receiving GC-free therapy
China (13)	a man in his thirties	Macular Edema, Behçet’s Uveitis	corticosteroids, methotrexate, cyclosporine A, mycophenolate mofetil and adalimumab	15mg	Ocular inflammation was clinically inactive, visual acuity improved and the therapy has been well-tolerated after upadacitinib used for nine months
China (Present case)	24, male	/	corticosteroids, infliximab, sulfasalazine, and adalimumab	45mg for 12 weeks and 30mg for 13weeks	Symptom relief, mucosal healing

Our findings contribute to the growing body of evidence supporting the use of selective JAK inhibitors in managing this difficult-to-treat condition. But the precise mechanisms underlying upadacitinib’s therapeutic effect in intestinal Behçet’s disease remain to be fully elucidated. Nonetheless, its targeted inhibition of JAK1 and JAK2, which are implicated in inflammatory pathways, may contribute to its ability to mitigate the excessive immune response seen in intestinal Behçet’s disease. The limitations of our case include the relatively short follow-up period and the solitary nature of the case. Further studies, including larger-scale clinical trials, are warranted to confirm the efficacy and safety of upadacitinib in intestinal Behçet’s disease and to define its optimal role in the therapeutic algorithm.

## Data Availability

The raw data supporting the conclusions of this article will be made available by the authors, without undue reservation.
